# Advances of magnetocardiography in application of adult and fetal cardiac diseases

**DOI:** 10.3389/fcvm.2025.1522467

**Published:** 2025-07-16

**Authors:** Junting Li, Yaqian Shen, Chengxing Shen, Xiaolin Ning, Min Xiang

**Affiliations:** ^1^ Key Laboratory of Ultra-Weak Magnetic Field Measurement Technology, Ministry of Education, School of Instrumentation and Optoelectronic Engineering, Beihang University, Beijing, China; ^2^ Department of Cardiology, Shanghai Sixth People's Hospital Affiliated to Shanghai Jiao Tong University School of Medicine, Shanghai, China; ^3^ State Key Laboratory of Traditional Chinese Medicine Syndrome / National Institute of Extremely-Weak Magnetic Field Infrastructure, Hangzhou, China; ^4^ Hefei National Laboratory, Hefei, China

**Keywords:** magnetocardiography, acute chest pain, ischemic heart disease, acute coronary syndrome, chronic cardiac disease, non-ischemic cardiomyopathy, arrhythmia, fetal magnetocardiography

## Abstract

Magnetocardiography (MCG) is a highly sensitive, non-invasive, and functional imaging technique that records and examines magnetic fields generated by the electrical activity of the heart to reflect cardiac electrophysiological changes, including the first superconducting quantum interference device and optically pumped magnetometers-MCG. The 60-year research process yields new understanding in the areas of signal extraction, processing, and clinical application for the detection and treatment of cardiac diseases. Especially, the significant advancements in magnetic sensor technology, preprocessing methods and denoising methods have promoted the development of MCG. This article systematically reviews 83 studies to provide the latest and general overview of MCG in acute chest pain (6 studies), acute coronary syndrome (10 studies), ischemic heart disease (13 studies), non-ischemic cardiomyopathies (3 studies), arrhythmia (9 studies), and fetal congenital arrhythmia (11 studies). We highlight its incremental value in the triage of acute chest pain, diagnosis and prognosis prediction of chronic and acute coronary syndromes. We also discuss the limitations of this field and directions of future development.

## 1 Introduction

Local currents are produced by depolarization, repolarization, and ion transmembrane mobility in cells. The electrophysiological activity of cells will produce weak magnetic signals, which are strongest in excitable cells, including myocardial and nerve cells. Magnetocardiography (MCG) can detect alterations in the magnetic fields induced by abnormal electrical activity in the heart through magnetically shielded or unshielded superconducting quantum interference device (SQUID) systems, optically pumped magnetometers (OPM), or portable miniaturized induction coils (1). Compared to electrocardiogram (ECG), which obtains electrical signals and current conventional imaging detection methods, MCG provides additional information for disease diagnosis through changes in magnetic signals and has unique advantages. First, MCG is not affected by the thickness of the pericardium or chest wall, providing more accurate information through its multi-channel array (2). In addition, MCG has a greater spatiotemporal resolution and is sensitive to the magnetic field produced by tangential current, which can simultaneously collect tangential and eddy currents in the subepicardial and deep myocardium (3–5).

Recent decades have seen numerous studies on signal processing and clinical applications of MCG. Several studies have concluded that MCG is more sensitive to triage of acute chest pain and diagnosis of ischemic heart disease (IHD) than widely used screening tools such as ECG, echocardiography (ECHO), and stress tests (6–8). Additionally, increasing research has linked MCG to fetal heart disease, myocardial inflammation, and arrhythmia. As an exceptionally sensitive, non-contact, non-invasive, and radiation-free examination method, MCG has potential clinical value. Therefore, we aimed to comprehensively summarize the literature on the clinical application of MCG and provide updated data.

## 2 Methods

In this review, we conducted an extensive electronic search of all English-language studies published before Oct 31, 2024, from the PubMed, Web of Science, EMBASE, and Cochrane Library databases. We used the following search terms: “magnetocardiography” AND (“heart disease” OR “cardiac disease” OR “fetal disease” OR “clinical” OR “chest pain” OR “coronary syndrome” OR “arrhythmia” OR “cardiomyopathy”). After removing books, editorials, case reports, letters and duplicates, we included studies that reported MCG in cardiac diseases. Furthermore, references cited in the included publications were examined to identify additional relevant articles. Finally, 83 studies published between January 1971 and October 2024 were included in this review.

## 3 Results

### 3.1 Magnetocardiography

The MCG technology has been developed for nearly 60 years. In 1970, MCG based on a SQUID was created (9). This magnetic detector offered high sensitivity, a wide measurement range, and a broad frequency band, significantly enhancing the spatial accuracy and signal-to-noise ratio (9). In the past 60 years, the development of multichannel sensors, unshielded SQUID systems, and the emergence of OPM have contributed to the advancement of MCG. While SQUID have been the primary tool for clinical research, OPM are being explored for their potential in making MCG more accessible. In 1991, Fenici et al. developed non-magnetic catheter technology based on 10 years of practical experience in MCG and successfully achieved arrhythmia localization through a single-channel system, laying early evidence for the clinical research and application of MCG (10).

At present, there is still no standardized MCG consensus or database to define the parameters of one-dimensional butterfly diagram (BFD), two-dimensional magnetic field map and current density map in MCG. Several scholars have recorded commonly utilized parameters of one-dimensional and multi-dimensional MCG, including interval duration, waveform, dipole phenomenon, and vector parameters, and have identified significant sex-based and age-related differences in amplitude and repolarization angle parameters (11, 12).

### 3.2 Rapid triage and mortality prediction of acute chest pain

Acute chest pain (ACP) continues a prevalent complaint in emergency and internal medicine, with over 7 million annual visits, of which 20%–40% are non-cardiac (13, 14). As a heterogeneous group of diseases, accurate and expeditious recognition of acute coronary syndrome (ACS), acute pulmonary embolism, aortic dissection, and other high-risk ACP conditions represents the primary focus and challenge in the emergency management of ACP. There is a lack of sensitive, convenient and rapid stratification methods to reduce the risk of misdiagnosis. Same as avoiding unnecessary invasive tests and minimizing patient loss.

Previous studies have investigated the utility of MCG in triaging patients with ACP (Table 1). Compared to conventional diagnostic tools (ECG, troponin I, and ECHO) at admission, MCG exhibited superior performance in identifying ACS and IHD through multiple MCG parameters including the angle, intensity, shape, and additional characteristics of the current or magnetic dipole vector during repolarization (6–8). Pena et al. developed a novel imaging and analysis system to transform MCG data into dynamic 90-second images to evaluate non-high-risk ACP patients in the emergency observation unit, its specificity (78.3%) and negative predictive value (NPV, 92.3%) suggested potential utility in safely ruling out critical ischemia and guiding discharge decisions (15). Ghasemi-Roudsari et al. further explored a portable MCG and built a diagnostic prediction model for ACP patients by logistic regression analysis of 10 key parameters derived from QR and RS segments in magnetic field map (MFM) and current density map (CDM), but the model showed limited accuracy in differentiating IHD among ACP patients (16). Beyond diagnosis, MCG parameters showed strong prognostic significance. Two MCG parameters of QTc prolongation and low repolarization reserve and one clinical parameter of elevated serum creatinine were indicators of long-term mortality in ACP patients (*P* < 0.05), achieving 90.9% sensitivity, 85.6% specificity, and 99.4% NPV for cardiac death (17). Patients with abnormal MCG findings faced a nine-fold increased risk of cardiac death (17).

**Table 1:** Studies of the diagnostic value of MCG in ACP.

Case	Study	Identified disease/Diagnostic criteria	Test condition	MCG parameters	Test group (*n*)/Control (*n*)	Tool	Sens (%)/ Spec (%)	NPV (%)/ PPV (%)	ROC AUC
1	Kwon et al. (6)	ACS/CA	Shielded 64-channel SQUID	ST interval: main current angle_max; ST interval: maximum of main current vector strength change; T_FMA; R_FMA	ACP suspected of ACS (364)/−	MCG	84.0/85.0	74.0/91.3	–
						ECG	44.7/89.8	46.5/89.1	–
					ACP suspected of ACS* (238)/−	MCG	78.1/82.6	70.4/87.7	–
						ECG	30.1/85.9	43.6/77.2	–
					ACP suspected of ACS† (181)/−	MCG	73.5/82.3	70.7/84.3	–
2	Park et al. (7)	ACS/CA	Unshielded 9-channel SQUID	T wave: main vector direction: −20° to +110°, and/or three subcriteria: angle changes over 45°; dipole distance over 20 mm; change between the plus and minus poles or strengths ratio over 0.3 within 30 ms.	ACP suspected of ACS (185)/−	MCG	95.1/92.8	84.8/97.8	–
						ECG	33.9/91.1	27.4/93.3	
						Troponin-I	42.7/90.5	31.7/93.8	
						ECHO	51.0/76.2	31.7/87.9	
3	Tolstrup et al. (8)	IHD/Troponin, stress testing or CA	At rest; Unshielded 9-channel SQUID	Automated quantitative EMDV score (one of angle, trajectory, angular deviation) in ventricular repolarization.	ACP (125)/−	MCG	76.4/74.3	80.0/70.0	–
						ECG	22.0/93.0	61.6/70.2	
4	Pena et al. (15)	ACS/Stress testing or CA	Unshielded 14-channel	Current or dipole deviations in T-wave	Non-high risk ACP (101)/−	MCG	33.3/78.3	92.3/13.0	–
5	Ghasemi-Roudsari et al. (16)	IHD/Magnetic resonance imaging scan, Myoview, or stress ECHO	Within 48 h or 4 weeks of chest pain; Unshielded portable prototype magnetometer	QR-peak; RS-peak; RS-MMR	IHD (70)/NIHD with ACP (69)	MCG	94.3/20.3	95.2/-	0.75
				QR-MMR; QR-angle; QR-pd; QR-peak; RS-MMR; RS-peak; RS-angle; RS-pd	IHD (70)/Healthy subjects (37)	MCG	100.0/78.4	100.0/-	0.96
				–	IHD (70)/ NIHD with ACP (69) and healthy subjects (37)	MCG	98.6/33.0	99.3/-	0.82

* Negative biomarker.

^†^ Negative biomarker and no specific ECG findings.

MCG, magnetocardiography; ACP, acute chest pain; Sens/Spec, sensitivity/specificity; NPV/PPV, negative predictive value/positive predictive value; ROC AUC, receiver operating characteristic/area under curve; CA, coronary angiography (stenosis ≥ 50% in at least one of coronary arteries was positive); SQUID, superconducting quantum interference device; ECG, electrocardiograph; FMA, magnetic field map angle; ECHO, echocardiography; EMDV, effective magnetic dipole vector; IHD, ischemic heart disease; MMR, amplitude ratio between the negative and positive pole; pd, distance between the negative and positive pole.

### 3.3 Acute coronary syndrome

ACS constitutes a continuous spectrum of life-threatening conditions initiated by coronary atherosclerotic plaque rupture and subsequent thrombotic occlusion, encompassing ST-elevation myocardial infarction (STEMI) and non-ST-elevation ACS (NSTE-ACS) (18). Characterized by high prevalence, sudden onset, and significant mortality, ACS requires urgent diagnosis to minimize the duration of emergency stay, as acute ischemia leads to irreversible myocardial cell necrosis. Currently, NSTE-ACS often lacks specific changes in ECG and myocardial damage indicators, requiring continuous testing for diagnosis. There is a pressing need for more efficient methods to facilitate early detection and risk stratification.

We compiled a comprehensive list of ACS articles pertaining to MCG (Table 2). In ACS, four qualitative and quantitative parameters including magnitude, angle, perimeter and area of MFM, as well as the T-wave CDM, exhibited marked abnormalities; T-peak maximum current angle and magnetic field angle had the highest sensitivity (96.4%), correlating positively with the severity of myocardial infarction (19–22). Among them, the integrated maximum CDM provided spatiotemporal information of ventricular repolarization process, with higher sensitivity (91.2%) and specificity (84.6%) for identifying STEMI than the integrated equivalent current dipole method (sensitivity, 84.3%; specificity, 82.1%) (19). ACS consistently exhibits non-dipole phenomena in MFM, with coherence impacted particularly during repolarization, the relationship of depolarization parameters and ACS was weak (23, 24). In 70% of unstable angina (UA) and 92.5% of non-ST-elevation myocardial infarction (NSTEMI), NSTE-ACS manifested as abnormal dipole position and shape, and was positively correlated with the severity of coronary artery stenosis (20).

**Table 2:** Studies of the diagnostic value of MCG in ACS.

Case	Study	Test group (*n*)/Control (*n*)	Test time and condition	Map type and parameters	Tool	Sens (%)/Spec (%)	NPV (%)/PPV (%)	ROC AUC
1	Leeuwen et al. (23)	Revascularized after STEMI (97)/Healthy subjects (39)	5.8 ± 3.0 days after infarction; Shielded 61-channel	MFM; STT interval	MCG	87.2/84.5	–	0.917
2	Lim et al. (20)	NSTEMI (83)/Young control (185) and age-matched control (19)	<3 days after hospital admission; Shielded 64-channel SQUID	MFM and CDM; 10 parameters from T wave and TT interval*	MCG	96.4/85.0	–	–
3	Zhao et al. (19)	STEMI (102)/Healthy subjects (39)	Shielded 61-channel SQUID	MFM and CDM; Magnitude, Angle, Perimeter and Area in T-wave interval	IECD	84.3/82.1	–	0.708–0.894
					IMCD	91.2/84.6	–	0.724–0.917
4	Goodacre et al. (27)	ACS (96)/Healthy subjects (584)	Portable VitalScan MCG	MCG algorithm and MACS clinical score	MCG	89.0/15.0	89.0/14.0	0.560
					MACS	–	–	0.690
					MCG + MACS	85.0/30.0	93.0/16.0	0.640
5	Mace et al. (24)	Suspected ACS and HEART score ≥3	Times of clinical convenience	MFM; QRS multipolarity, T wave multipolarity, RT angle, T wave dynamics, and ST segment abnormalities	MCG	66.7/57.1	–	–

* TT interval: interval from Tmax/3 to Tmax.

MCG, magnetocardiography; ACS, acute coronary syndrome; Sens/Spec, sensitivity/specificity; NPV/PPV, negative predictive value/positive predictive value; ROC AUC, receiver operating characteristic/area under curve; STEMI, ST-segment elevation myocardial infarction; NSTEMI, non-ST-segment elevation myocardial infarction; SQUID, superconducting quantum interference devices; MFM, magnetic field maps; CDM, current density map; IECD, Integrated equivalent current dipole; IMCD, integrated maximum current density; MACS, manchester acute coronary syndrome.

MCG also demonstrated predictive capacity for clinical outcomes. Bang et al. illustrated that non-dipole phenomenon of T-peak independently predicted major adverse cardiac events, including all-cause death, reinfarction, and percutaneous coronary intervention (hazard ratio = 2.89, 95% confidence interval: 1.20–6.97, *P* = 0.02) (25). A three-year follow-up study of NSTEMI patients revealed that MCG outperformed troponin I, ECHO, and ECG in mortality prediction (relative risk: 4.58 vs. 2.48 vs. 1.58 vs. 1.69), with abnormal MCG independently portending poor prognosis (7, 26).

It can be seen that MFM and CDM during the repolarization phase have application prospects for both diagnosis and prognostic prediction in ACS. Future research requires further quantification of MFM and CDM parameters to establish a unified standard. Additionally, MCG has only been exclusively detected after the treatment of acute myocardial infarction, and point-of-care device reliability has not yet been fully validated (27). Further investigations are warranted to examine the feasibility of early detection in the post-event period.

### 3.4 Detection and classification of ischemic heart disease

IHD arises from a disparity between coronary blood supply and myocardial oxygen demand, caused by coronary spasm, stenosis, or obstruction, which ultimately leads to myocardial hypoxia or necrosis. IHD manifests as angina, myocardial infarction or even sudden cardiac death clinically. As a major global health concern, IHD affects 18.2 million cases in the United States alone and remains the leading cause of death worldwide, with 9.24 million global deaths attributed to IHD in 2022, representing 80% of sudden cardiac fatalities and 34% of cardiovascular disease-related deaths under before aged 70 (28–30). The heterogeneous clinical presentations and complex differential diagnosis of IHD pose significant challenges in developing rapid and accurate methods for case identification and risk stratification.

ECG and ECHO serve as first-line screening tools for IHD detection. A meta-analysis of 147 studies involving 24,074 patients reported a sensitivity of 68% and specificity of 77% for ECG stress tests (31). Coronary computed tomography angiography (CCTA) offers a high diagnostic accuracy in assessing anatomic coronary stenosis, with a sensitivity of 94%, specificity of 83%, and NPV of 99% (32, 33). However, its poor positive predictive value (PPV) (48%–64%) and inability to reliably evaluate hemodynamic changes or screen large-scale population necessitate additional supplementary testing to determine ischemia severity (33, 34). Existing non-invasive functional tests use perfusion imaging with radioactive tracers to evaluate myocardial viability, though these methods carry inherent risks of radiation exposure and potential adverse effects. In contrast, MCG demonstrates promising diagnostic performance. Three studies have shown that abnormal ST-segment and T-wave in MCG during myocardial ischemia can be correctly classified in 80% of cases using only one parameter (sA4), irrespective of the number or location of affected vessels, when combining all three parameters achieved 84% sensitivity and 83% specificity (area under the curve, AUC: 0.912) (3, 35). Comparative analyses revealed that MCG exhibited greater mean differences in QRS complex, T-wave, and ST-segment abnormalities than ECG (37% vs. 26%) (36).

We visually listed the literature on the diagnostic value of MCG for coronary stenosis in Table 3. Whether at rest or under stress testing, MCG demonstrated superior efficacy than ECG in identifying chronic coronary disease (CCD), regardless of whether recordings were obtained in magnetically shielded or unshielded environments (37–40). Additionally, QTc parameters of MCG successfully screened CCD patients at rest, providing a viable alternative for individuals unable to tolerate stress testing (41). Machine learning-based MCG diagnostic models have achieved high sensitivity (82.6%–91.3%) in detecting myocardial ischemia, though specificity remains modest (10.0%–50.0%) (42). For borderline coronary lesions (40%–90% stenosis), MCG yielded an AUC of 0.864 (95% CI: 0.803–0.925), suggesting its potential to reduce unnecessary invasive procedures (43).

**Table 3:** Studies of the diagnostic value of MCG in CCD.

Case	Study	Test group (*n*)/Control (*n*)	Test condition	MCG maps and parameters	Tool	Sens (%)/ Spec (%)	NPV (%)/ PPV (%)	ROC AUC
1	Gapelyuk et al. (3)	CCD (101)/Healthy subjects (59)	At rest; Shielded 7-channel SQUID	sA4	MCG	79.0/73.0	–	–
				sA6		84.0/81.0	–	–
				sA4, sA6, *Δ*MFM orientation		84.0/83.0	–	0.912
2	Park et al. (37)	Suspected CCD (100)/−	At rest and dobutamine-atropine scheme	Current strength/density	MCG	97.6/82.8	–	–
					ECG	26.2/82.8	–	–
3	Fenici et al. (38)	Stable angina (51)/Healthy subjects (52)	At rest; unshielded 36-channel SQUID	Current density map; T-wave dynamics and effective magnetic vector parameters	MCG	83.0/96.0	89.0/94.0	–
					ECG	38.0/100.0	80.0/100.0	–
4	Shin et al. (39)	Suspected CCD (202)/−	At rest and exercise; Shielded 64-channel SQUID	Butterfly diagram; ST-segment fluctuation score: −40.0%	Exercise MCG	68.4/95.1	82.4/90.0	0.839
					Exercise ECG	40.5/91.1	70.4/74.4	0.658
5	Wu et al. (41)	CCD (36)/Healthy subjects (19)	At rest; Shielded 64-channel SQUID	QTc dispersion ≥ 79 ms	MCG	83.3/68.4	–	0.758
				Smooth index-QTc ≥ 9.1 ms	MCG	77.8/68.4	–	0.731
				Combination of the above two	MCG	86.1/68.4	–	0.773
				Stress nuclear MPI	MPI	69.4/94.7	–	0.820
6	Shin et al. (44)	Suspected CCD (96)/-	At rest and stress (bicycle exercise test); Shielded 64-channel SQUID	ST-segment fluctuation score: −51.0%	MCG	73.9/82.0	77.4/79.1	0.790
				Non-dipole phenomenon in T wave	MCG	84.8/88.0	86.3/86.7	0.864
				Incorporation of the above two	MCG	–	–	0.930
7	Park et al. (45)	Suspected CCD (47)/-	At rest and stress (exercise and dobutamine stress test); Shielded 64-channel SQUID	ST-segment fluctuation score: −39.0%	MCG	86.7/73.9	–	0.835
8	Ramesh et al. (46)	TMT + (12)/ TMT- (17)	normal ECG; Shielded 37-channel SQUID	Magnetic field map; magnetic field angle (normal: between −86° and −45°) at T-wave peak	Magnetic angle	81.8/94.1	–	–
					Magnetic map	81.8/94.1		
					Either one	90.9/94.1		
9	Hailer et al. (47)	CCD (177)/ Healthy group (117)	Normal ECG; At rest; Unshielded 4-channel SQUID	Current density vector maps during ST-T interval	MCG	73.3/70.1	–	–
		nCCD (123)/ Healthy group (117)				73.9/49.6		
		CCD (177)/ nCCD (123)				62.8/61.3		
10	Chaikovsk et al. (48)	CCD (54)/Chest pain without stenosis (25)	Unshielded 7-channel	Complex index: AIQRS_total_, AIST-T_total_, A_dur_, C_cor_, R/T_current_, and MAP_typ_	MCG	93.0/84.0	93.0/85.0	–
		CCD (54)/Healthy subjects (30)			MCG	93.0/94.0	93.0/94.0	–
11	Shin et al. (49)	CCD (35)/nCCD (73)	At rest and bicycle exercise test; Shielded 9-channel SQUID	Complex index: Positive T-wave score at stress, T-wave dispersion at stress, T-wave VMCG at rest, %change of T-wave VMCG, %change of 1/2RT-interval VMCG	MCG	89.0/77.0	91.0/74.0	0.91
12	Cui et al. (50)	sCS (406)/nsCS (107)	At rest; Unshielded 9-channel SQUID	Butterfly diagram and magnetic field map: QR_MCTDd; QR_MVamp; R_MA; S_MA; S_MDp; T_MA; TT_MAC50	MCG	71.7/80.4	42.8/93.3	0.810
					MCG, T2DM and Apoprotein A1	84.3/73.8	54.6/92.6	0.845
					Magnetic map	81.8/94.1	–	–
13	Huang et al. (51)	CCD (128)/ nCCD (81)	Unshielded 4-channel SQUID	10 parameters of T wave: current angle, field map angle, distance	MLP	91.4/87.7	86.6/92.1	0.954
14	Tantimongcolwat et al. (52)	IHD (55)/Healthy subjects (70)	9-channel SQUID	J-T interval	BNN	89.7/54.5	–	–
					DK-SOM	86.2/72.7	–	–
15	Kangwanariyakul et al. (53)	IHD (55)/Healthy subjects (70)	36-channel SQUID	J-T interval	BPNN	86.2/68.1	–	0.905
					BNN	96.6/54.5	–	0.849
					Polynomial SVM	89.6/45.4	–	–
					RBF SVM	41.3/86.3	–	–
16	Steinisch et al. (54)	CCD (4)/nCCD with ACP (6)	At rest, during pharmacological stress and during recovery; 61-channel	QRS complex and ST-T segment; MLP based on LDA	At rest	99.0/97.4	99.4/96.0	–
					During stress	86.2/60.0	88.0/56.2	–
					During recovery	71.8/83.7	82.3/73.8	–
17	Rong et al. (55)	CCD (227)/Healthy subjects (347)	Unshielded 4-channel (MD-U041001)	164 features in T wave: time domain (18), frequency domain (108), information theory (38)	SVM-XGBoost mixed model	−/94.0	–	0.98
18	Zhang et al. (42)	Impaired myocardial perfusion (70)/Normal myocardial perfusion (42)	36-channel OPM-MCG	Five parameters	RF	87.0/50.0	62.5/80.0	0.80
					DT	82.6/30.0	42.8/73.0	0.78
					SVM	91.3/10.0	33.3/70.0	0.80

MCG, magnetocardiography; CCD, chronic coronary disease; Sens/Spec, sensitivity/specificity; NPV/PPV, negative predictive value/positive predictive value; ROC AUC, receiver operating characteristic/area under curve; CA, coronary angiography; ECG, electrocardiography; SQUID, superconducting quantum interference device; sA4, ST slope for location A4; sA6, ST slope for location A6; MFM, magnetic field map; SPECT, single-photon emission computed tomography; QTc, QT/(R-R)1/2; TMT, treadmill test; nCCD, non-chronic coronary disease; AIQRStotal, the mean value of AI during the QRS complex; AIST-Ttotal, the mean value of AI during the ST-T interval; Adur, Ccor, R/Tcurrent, the total current ratio between the R and the T peak; MAPtyp, normality degree of maps’ for the myocardial ischemia; VMCG, vector magnetocardiography; sCS, severe coronary stenosis (≥70%); nsCS, non-severe coronary stenosis; MCTDd, distribution of magnetic dipole center trajectory; MVamp, magnetic pole vector based on amplitude; MA, magnetic pole angle; MDp, dispersion of magnetic pole; MAC50, angle changes of magnetic pole; T2DM, type 2 diabetes mellitus; MLP, multilayer perceptron; IHD, ischemic heart disease; BNN, Bayesian neural network; DK-SOM, direct kernel self-organizing map; BPNN, back propagation neural network; SVM, support vector machine; RBF, radial basis function; ACP, acute coronary pain; LDA, linear discriminant analysis; SVM-XGBoost, support vector machine-eXtreme gradient boosting; OPM-MCG, optically pumped magnetometers-magnetocardiogarphy; RF, random forest; DT, decision tree.

Different MCG parameters and analytical approaches have been explored to optimize coronary stenosis detection in suspected CCD patients. The combination of ST-segment fluctuation score and qualitative non-dipole parameter achieved the highest diagnostic accuracy (AUC: 0.93) (39, 44, 45). In chronic chest pain patients with normal ECG, magnetic field map and angles distinguished coronary stenosis with 90.9% sensitivity and 94.1% specificity (46). CDM during ST-T interval effectively differentiated healthy individuals from CCD patients, though their ability to distinguish non-CCD from CCD cases was inferior to MFM (47). Integrating one-dimensional BFD with MFM enhanced diagnostic performance and localized stenosis with 69%–77% accuracy (48–50). To expedite data processing and minimize manual errors, automated machine learning approaches are applied for parameter extraction and analysis. Bayesian neural networks and multilayer perceptron demonstrated high sensitivity, making them suitable for high-risk population screening (51–54). A hybrid classifier Support Vector Machine-eXtreme Gradient Boosting, incorporating 164T-wave features (time domain, frequency domain, and information theory characteristics), achieved exceptional performance (AUC, 0.98; specificity, 94.0%) while effectively reducing false positives (55). In summary, MCG showed particular strengths in identifying ischemia in resting patients and those with borderline lesions. Individual parameters in MFM and CDM as well as the complex index in BFD are all strong predictors, AI-enhanced approaches further improved diagnostic accuracy. Notably, MCG also showed acceptable potential for detecting non-obstructive coronary microvascular dysfunction (sensitivity: 68%, specificity: 65%) (56).

It is important to emphasize that all aforementioned studies were conducted on patients with CCD, indicating that MCG can detect lesions with coronary stenosis of ≥70%. However, for vascular stenosis that has not yet affected the structure or function of cells, it does not cause abnormal conduction or magnetic signals, MCG will be unable to detect it. In addition, the relationship between the degree of coronary stenosis and changes in magnetocardiographic vector has not yet been standardized, so the current evidence supporting magnetocardiographic evaluation in this context remains limited and can not contribute to clinical decision-making.

### 3.5 Non-ischemic cardiomyopathies

Beyond IHD, a spectrum of cardiac disorders arising from genetic predisposition, metabolic dysfunction, and structural myocardial changes are collectively termed non-ischemic cardiomyopathies (57). Integrating more dimensions to achieve precise classification of cardiomyopathies is a way forward for efficient diagnosis and treatment. Emerging research have investigated the promising role of MCG in the diagnosis, treatment monitoring, and recurrence prediction of non-ischemic cardiomyopathies.

Changes of magnetic vector (the value ≥ 0.051) discriminated cardiomyopathy from controls with 59% sensitivity, 95% specificity, 93% PPV, and 64% NPV, and reflected immune-suppressive therapeutic effect earlier than ECHO (7 vs. 30 days) (58). In dilated cardiomyopathy, MCG effectively predicted major adverse cardiac events (MACE) by detecting left intraventricular disorganized conduction (LIDC) with high spatiotemporal resolution to facilitate risk stratification, and the predictive value of LIDC was superior to that of traditional ECG indices such as fragmented QRS waves and late potential (59). By capturing early changes of myocardial electrical remodeling and quantifying the Kullback Leibler (KL) entropy of the cardiac magnetic field topology, MCG differentiated hypertrophic cardiomyopathy from healthy individuals or cardiac hypertrophy caused by other reasons, and the accuracy increased to 87.9% when combined with regional magnetic field strength parameters (sensitivity from 78.8% to 84.8% and specificity from 86.9% to 88.9%), and identified all patients with hypertrophic cardiomyopathy carrying genetic mutation in familial screening (60).

### 3.6 Diagnosis and localization of arrhythmia

Compared to ECG, MCG has a enhanced spatial resolution of cardiac current distribution and more comprehensive insights into ventricular depolarization and repolarization. As summarized in Table 4, growing evidence supports the utility of MCG in risk stratification, original localization, and management guidance.

**Table 4:** Studies of the diagnostic value of MCG in cardiac arrhythmia.

Case	Study	Test group (n)/Control (n)	Test time and condition	Map type and parameters	Tool	Sens (%)/Spec (%)	NPV (%)/PPV (%)	ROC AUC
1	Iwakami et al. (61)	ERP-VF + (13)/ERP-VF- (103)	After survived the VF; Shielded 64-channel SQUID	MCG: current arrow map; QRSD, RMS40, LAS. ECG: ST morphology, T-wave/R-wave (T/R) ratio, J-wave distribution or configuration, J-peak amplitude.	QRSD ≥ 100 ms	69.0/74.0	–	0.72
					RMS40 ≤ 0.24	92.0/48.0	–	0.71
					ECG parameters	8.0–85.0/10.0–93.0	–	0.50
2	Her et al. (66)	Paroxysmal atrial fibrillation (22)/Healthy subjects (26) and marathon runners (22)	At rest and stress (bicycle exercise test); Shielded 64-channel SQUID	STAG; Peak value of PQ fluctuation score, LA pseudo-current increase, PQ mapping STAG.	MCG	76.7/91.7	97.3/50.6	0.896
3	Ito et al. (68)	VA from RVOT (41) or ASC (10)/−	One day before ablation; Shielded 64-channel MCG	3-D MCG imaging; Depth of the VA origin relative to the sensor array, distance between sinus node and origin of VA, magnetic field orientation at the QRS complex peak of the VA signal.	The depth of origin	90.0/73.0	41/97	0.90
4	Aita et al. (69)	Drug-refractory premature ventricular contractions (22)/−	Before ablation; Shielded 64-channel SQUID	3-D current distribution of the heart.	MCG-CT mapping	94.0	–	–
					ECG algorithms	56.0	–	–

MCG, magnetocardiography; Sens/Spec, sensitivity/specificity; NPV/PPV, negative predictive value/positive predictive value; ROC AUC, receiver operating characteristic/area under curve; ERP-VF, early repolarization pattern-ventricular fibrillation; SQUID, superconducting quantum interference devices; QRSD, QRS duration; RMS40, root-mean-square of the last 40 ms; LAS, low amplitude (<10% of maximal) signal duration; STAG, spatiotemporal activation graph; LA, left atrial; VA, ventricular arrhythmia; RVOT, right ventricular outflow tract; ASC, aortic sinus cusp; MCG-CT, magnetocardiography-computed tomography.

There was no statistical difference in ECG parameters between benign and malignant early repolarization patterns (ERP). Conversely, ERP-ventricular fibrillation (ERP-VF) patients in MCG had prolonged QRS complex (108 ± 24 vs. 91 ± 23 ms, *P* = 0.02) and diminished root-mean-square voltage of the last 40 ms (0.10 ± 0.08 vs. 0.25 ± 0.20, *P* = 0.01) (61). In addition, compared with invasive electrophysiological mapping, the accuracy of MCG in differentiating benign and malignant ventricular arrhythmia was 94.7% (62). Beyond non-invasive risk stratification for VF, the low-amplitude QRS complex also predicted arrhythmia risk after MI, with QRS duration > 121 ms as an independent predictor (63, 64). The P-wave duration, PR interval and P-wave depolarization of patients with atrial fibrillation were significantly longer than those of healthy controls, providing more sensitive atrial fibrillation susceptibility markers than ECG, the angle dynamics of P-wave depolarization was an independent predictor of AF recurrence (*P* = 0.037) (65). The alteration in left atrial pseudo-current conversion detected left atrial dysfunction in paroxysmal atrial fibrillation, and identify different atrial conduction pathways (such as Bachmann bundles, margin of fossa ovalis, and coronary sinus ostial region) with an accuracy of 93%, providing a new perspective for the mechanism study of atrial fibrillation (66, 67).

The multi-channel sensors of MCG provide insights into the temporal and spatial distribution of cardiac magnetic fields and enable the localization of arrhythmia origins through three-dimensional imaging. Ito et al. developed a new spatial filter to differentiate ventricular arrhythmia originating from the right ventricular outflow tract and the aortic sinus cusp, through three parameters, with depth being the most powerful predictor (68). By integrating cardiac computed tomography to reconstruct the 3-D current distribution, the origin of premature ventricular complexes throughout the ventricle was pinpointed with 94% accuracy (17/18) (69).

### 3.7 Fetal magnetocardiography

Current evaluation of fetal cardiac structure and function relies on cardiotocography and ECHO, but they lack electrophysiological data on the conduction system and cannot identify certain malignant arrhythmia such as torsade de pointes (70). Fetal MCG (fMCG) allows for precise assessment of cardiac time intervals, signal characteristics, and various rhythm patterns by extracting fetal cardiac magnetic signals after the 15 weeks of pregnancy (71–74). It provides vital information about fetal cardiac development and function, as shown in Table 5.

**Table 5:** Studies of the diagnostic value of fMCG.

Case	Study	Study group (n, GA)	Control group (n, GA)	Testing conditions	fMCG waveform features (study group vs. control group)
1	Cuneo et al. (79)	Suspected LQTS (30, 19–38)		Shielded 37-channel and 64-channel biomagnetometers	LQTS: QTc > 490 ms; late-peaking T-wave morphology; TdP: QTc ≥ 620 ms, with more complex waveforms
2	Kiefer-Schmidt et al. (80)	Exposed to SSA/Ro- or SSB/La antibodies (11, 20–37)	Healthy (87, 17–41)	156-channel biomagnetic system	PQ segments: 50.8 ms vs. 60.2 ms; *P* < 0.001
3	Zhao et al. (81)	Second-degree AVB (5, 19–32)	–	Shielded 37-channel biomagnetometer	Complex, changing rhythms, e.g., intermittent pre-excitation, alternating high and low atrial rhythms, and variable AV conduction.
		Third-degree AVB (23, 20–32)	–		Junctional ectopic tachycardia and ventricular tachycardia
4	Wiggins et al. (83)	BAB (10, 21–29.3)	–	Shielded 37-channel axial gradiometer or 21-channel vector	Ectopic P wave (P’) occurred early and heart rate was faster. PP′/PP = 0.29 ± 0.03, PP′ = 209 ± 23 ms, FHR = 82 ± 5.7 beats/min
		Second-degree type II AVB (5, 25–37.2)	–		PP′/PP = 0.49 ± 0.01, PP′ = 419 ± 50.5 ms, FHR = 69 ± 4.2 beats/min

fMCG, fetal magnetocardiography; GA, gestational age; LQTS, long QT syndrome; TdP, torsades de pointes; AVB, atrioventricular block; BAB, blocked atrial bigeminy; FHR, fetal heart rate; PP, interval from the P wave of the sinus beat to the P wave of the premature beat.

Both the 2014 and 2024 American Heart Association scientific statements advocated fMCG to assess cardiac conduction and rhythm abnormalities in fetuses with suspected or confirmed congenital heart disease (Class 2a) (75, 76). Strand et al. used SQUID to characterize fMCG waveforms of 132 healthy fetuses at 15.7–39.9 weeks of gestation, P-wave, PR-interval, QRS complex, and RR interval increased with gestational age (*P* < 0.001), while QT-interval and QTc (QTc = QT/RR1/2) remained constant throughout gestation (73). In contrast, another study used new magnetograph dedicated to fetal recordings and concluded that P wave, QRS complex, ST-segment, QT-interval and QTc increased with gestational age, PQ segment and T-wave were independent of gestation (77). It is imperative to elucidate the waveform characteristics of fetuses at different developmental stages.

Wacker-Gussmann et al. reviewed 144 fetuses with tachycardia, bradycardia atrioventricular block (AVB), or familial Long QT syndrome (LQTS) (71). As a result, fMCG facilitated additional diagnoses in 81% (117/144) of cases, with 56% (81/144) exhibiting critical alterations, prompting a change in the treatment regimen for 35 patients (71). LQTS is a hereditary ion channel disorder that leads to potentially fatal heart rhythm abnormalities and accounts for 10% of sudden infant death and unexplained stillbirths (78). Early detection of abnormal heart rates and intrauterine treatment can reduce mortality. Research published in Circulation in 2013 showed that QTc > 490 ms, corroborated by genetic testing, achieved 89% sensitivity and specificity for LQTS between 19 and 38 weeks of gestation, with QTc ≥ 620 ms predicting a severe TdP phenotype, characterized by various uncommon rhythms such as second-degree AVB, T-wave alternation, and QRS alternation (79).

The waveform characteristics of AVB with different degrees of severity are much more complex than those of ECHO. Fetuses with positive maternal SSA/Ro SSB/La antibodies are at elevated risk of developing immune AVB. The prolonged PQ segment predicts early atrioventricular node involvement in fetuses (60.2 ms vs. 50.8 ms; *P* < 0.001) (80). Different waveforms classified AVB and accurately identified third-degree AVB with structural heart disease and poor prognosis (81, 82). ECHO struggled to distinguish blocked atrial bigeminy (BAB) from 2nd degree AVB, despite their divergent clinical management and prognosis (83). fMCG discerned differences between the two entities, with the ectopic P wave (P’) manifesting earlier and a higher heart rate observed in BAB cases, thus offering pivotal evidence for differential diagnosis (83).

## 4 Limitations

Although many studies have proved that MCG has excellent clinical diagnostic and prognostic predictive capabilities across various cardiac diseases and is a promising clinical tool in the future, certain limitations persist within this domain. First, there exists heterogeneity in study design, and parameters, with different methodologies, MCG systems, and analytical parameters employed across studies. The absence of standardized protocols in the acquisition of cardiomagnetic images and data, as well as in parameter definitions and diagnostic thresholds constrains the general applicability and clinical advancement of research findings. Establishing uniform guidelines for MCG interpretation is crucial for advancing the field. Secondly, most clinical studies of MCG are conducted in single-center with small cohorts and meticulously screened populations. This may introduce selection bias and lead to an overestimation of diagnostic accuracy. Consequently, larger, multi-center studies are necessary to validate the reproducibility of MCG across diverse populations, devoid of established clinical backgrounds and in varied environments. Finally, research evidence concerning non-ischemic cardiomyopathy and UA is limited, and there are few studies on MCG parameters and long-term prognosis verification, thereby limiting the robustness of MCG results. Therefore, future research must focus on optimizing the standardization of MCG data collection processes, establishing unified parameter reference ranges, and identifying specific parameters for various diseases.

## 5 Conclusion

Magnetocardiography provides a new perspective for the rapid and non-invasive identification of critical and severe heart diseases. The high spatial and temporal resolution and absence of interference from human tissue with the magnetic signal confer excellent functional imaging capabilities to MCG, demonstrating superior diagnostic performance for adult and fetal heart disease, including MI, adult and fetal arrhythmia, and rapid triage of ACP. However, its application is still limited to a few research centers, and the lack of uniform equipment standards and specifications makes it difficult to directly compare the results between different devices. Furthermore, there remains a paucity of large-scale clinical evidence and well-defined diagnostic criteria for MCG.

Future research should focus on validating parameters, establishing usage standards, and integrating MCG into clinical practice to develop high-performance devices suitable for routine environments, ensuring the rapid and accurate diagnosis of acute and severe heart diseases.

## References

[1] Wang Y Zhao ZG Chai Z Fang JC Chen M. Electromagnetic field and cardiovascular diseases: a state-of-the-art review of diagnostic, therapeutic, and predictive values. FASEB J. (2023) 37(10):e23142. 10.1096/fj.202300201RR 37650634

[2] Plonsey R. Capability and limitations of electrocardiography and magnetocardiography. IEEE Trans Biomed Eng. (1972) 19(3):239–44. 10.1109/TBME.1972.324123 5021223

[3] Gapelyuk A Wessel N Fischer R Zacharzowsky U Koch L Selbig D Detection of patients with coronary artery disease using cardiac magnetic field mapping at rest. J Electrocardiol. (2007) 40(5):401–7. 10.1016/j.jelectrocard.2007.03.013 17531250

[4] Dutz S Bellemann ME Leder U Haueisen J. Passive vortex currents in magneto- and electrocardiography: comparison of magnetic and electric signal strengths. Phys Med Biol. (2006) 51(1):145–51. 10.1088/0031-9155/51/1/011 16357437

[5] Smith FE Langley P van Leeuwen P Hailer B Trahms L Steinhoff U Comparison of magnetocardiography and electrocardiography: a study of automatic measurement of dispersion of ventricular repolarization. Europace. (2006) 8(10):887–93. 10.1093/europace/eul070 16837488

[6] Kwon H Kim K Lee YH Kim JM Yu KK Chung N Non-invasive magnetocardiography for the early diagnosis of coronary artery disease in patients presenting with acute chest pain. Circ J. (2010) 74(7):1424–30. 10.1253/circj.CJ-09-0975 20508380

[7] Park JW Hill PM Chung N Hugenholtz PG Jung F. Magnetocardiography predicts coronary artery disease in patients with acute chest pain. Ann Noninvasive Electrocardiol. (2005) 10(3):312–23. 10.1111/j.1542-474X.2005.00634.x 16029382 PMC6932599

[8] Tolstrup K Madsen BE Ruiz JA Greenwood SD Camacho J Siegel RJ Non-invasive resting magnetocardiographic imaging for the rapid detection of ischemia in subjects presenting with chest pain. Cardiology. (2006) 106(4):270–6. 10.1159/000093490 16733351

[9] Cohen D Norman JC Molokhia F Hood W Jr. Magnetocardiography of direct currents: S-T segment and baseline shifts during experimental myocardial infarction. Science. (1971) 172(3990):1329–33. 10.1126/science.172.3990.1329 5580214

[10] Fenici RR Melillo G Masselli M. Clinical magnetocardiography. 10 years experience at the catholic university. Int J Card Imaging. (1991) 7(3-4):151–67. 10.1007/BF01797748 1820397

[11] Kandori A Ogata K Watanabe Y Takuma N Tanaka K Murakami M Space-time database for standardization of adult magnetocardiogram-making standard MCG parameters. Pacing Clin Electrophysiol. (2008) 31(4):422–31. 10.1111/j.1540-8159.2008.01011.x 18373760

[12] Sorbo AR Lombardi G La Brocca L Guida G Fenici R Brisinda D. Unshielded magnetocardiography: repeatability and reproducibility of automatically estimated ventricular repolarization parameters in 204 healthy subjects. Ann Noninvasive Electrocardiol. (2018) 23(3):e12526. 10.1111/anec.12526 29266621 PMC6931803

[13] Kontos MC de Lemos JA Deitelzweig SB Diercks DB Gore MO Hess EP 2022 ACC expert consensus decision pathway on the evaluation and disposition of acute chest pain in the emergency department: a report of the American college of cardiology solution set oversight committee. J Am Coll Cardiol. (2022) 80(20):1925–60. 10.1016/j.jacc.2022.08.750 36241466 PMC10691881

[14] Eslick GD Jones MP Talley NJ. Non-cardiac chest pain: prevalence, risk factors, impact and consulting–a population-based study. Aliment Pharmacol Ther. (2003) 17(9):1115–24. 10.1046/j.1365-2036.2003.01557.x 12752348

[15] Pena ME Pearson CL Goulet MP Kazan VM DeRita AL Szpunar SM A 90-second magnetocardiogram using a novel analysis system to assess for coronary artery stenosis in emergency department observation unit chest pain patients. Int J Cardiol Heart Vasc. (2020) 26:100466. 10.1016/j.ijcha.2019.100466 31956695 PMC6956743

[16] Ghasemi-Roudsari S Al-Shimary A Varcoe B Byrom R Kearney L Kearney M. A portable prototype magnetometer to differentiate ischemic and non-ischemic heart disease in patients with chest pain. PLoS One. (2018) 13(1):e0191241. 10.1371/journal.pone.0191241 29351337 PMC5774725

[17] Wessel N Kim JS Joung BY Ko YG Dischl D Gapelyuk A Magnetocardiography at rest predicts cardiac death in patients with acute chest pain. Front Cardiovasc Med. (2023) 10:1258890. 10.3389/fcvm.2023.1258890 38155993 PMC10752986

[18] Byrne RA Rossello X Coughlan JJ Barbato E Berry C Chieffo A 2023 ESC guidelines for the management of acute coronary syndromes. Eur Heart J. (2023) 44(38):3720–826. 10.1093/eurheartj/ehad191 37622654

[19] Zhao C Jiang S Wu Y Zhu J Zhou D Hailer B An integrated Maximum current density approach for noninvasive detection of myocardial infarction. IEEE J Biomed Health Inform. (2018) 22(2):495–502. 10.1109/JBHI.2017.2649570 28092581

[20] Lim HK Kwon H Chung N Ko YG Kim JM Kim IS Usefulness of magnetocardiogram to detect unstable angina pectoris and non-ST elevation myocardial infarction. Am J Cardiol. (2009) 103(4):448–54. 10.1016/j.amjcard.2008.10.013 19195500

[21] Lim HK Chung N Kim K Ko YG Kwon H Lee YH Can magnetocardiography detect patients with non-ST-segment elevation myocardial infarction? Ann Med. (2007) 39(8):617–27. 10.1080/07853890701538040 17852033

[22] Lim H K Kim K Lee YH Chung N. Detection of non-ST-elevation myocardial infarction using magnetocardiogram: new information from spatiotemporal electrical activation map. Ann Med. (2009) 41(7):533–46. 10.1080/07853890903107883 19626486

[23] Van Leeuwen P Hailer B Beck A Eiling G Grönemeyer D. Changes in dipolar structure of cardiac magnetic field maps after ST elevation myocardial infarction. Ann Noninvasive Electrocardiol. (2011) 16(4):379–87. 10.1111/j.1542-474X.2011.00466.x 22008494 PMC6932076

[24] Mace SE Peacock WF Stopyra J Mahler SA Pearson C Pena M Accelerated magnetocardiography in the evaluation of patients with suspected cardiac ischemia: the MAGNETO trial. Am Heart J Plus. (2024) 40:100372. 10.1016/j.ahjo.2024.100372 38586432 PMC10994860

[25] Bang WD Kim K Lee YH Kwon H Park Y Pak HN Repolarization heterogeneity of magnetocardiography predicts long-term prognosis in patients with acute myocardial infarction. Yonsei Med J. (2016) 57(6):1339–46. 10.3349/ymj.2016.57.6.1339 27593860 PMC5011264

[26] Park JW Leithäuser B Hill P Jung F. Resting magnetocardiography predicts 3-year mortality in patients presenting with acute chest pain without ST segment elevation. Ann Noninvasive Electrocardiol. (2008) 13(2):171–9. 10.1111/j.1542-474X.2008.00217.x 18426443 PMC6932191

[27] Goodacre S Walters SJ Qayyum H Coffey F Carlton E Coats T Diagnostic accuracy of the magnetocardiograph for patients with suspected acute coronary syndrome. Emerg Med J. (2021) 38(1):47–52. 10.1136/emermed-2020-210396 33051274

[28] Mensah GA Fuster V Roth GA. A heart-healthy and stroke-free world: using data to inform global action. J Am Coll Cardiol. (2023) 82(25):2343–9. 10.1016/j.jacc.2023.11.003 38092508

[29] Mensah GA Fuster V Murray CJL Roth GA. Global burden of cardiovascular diseases and risks, 1990–2022. J Am Coll Cardiol. (2023) 82(25):2350–473. 10.1016/j.jacc.2023.11.007 38092509 PMC7615984

[30] GBD 2021 Causes of Death Collaborators. Global burden of 288 causes of death and life expectancy decomposition in 204 countries and territories and 811 subnational locations, 1990–2021: a systematic analysis for the global burden of disease study 2021. Lancet. (2024) 403(10440):2100–32. 10.1016/S0140-6736(24)00367-2 38582094 PMC11126520

[31] Detrano R Gianrossi R Froelicher V. The diagnostic accuracy of the exercise electrocardiogram: a meta-analysis of 22 years of research. Prog Cardiovasc Dis. (1989) 32(3):173–206. 10.1016/0033-0620(89)90025-X 2530605

[32] Sirajuddin A Mirmomen SM Kligerman SJ Groves DW Burke AP Kureshi F Ischemic heart disease: noninvasive imaging techniques and findings. Radiographics. (2021) 41(4):990–1021. 10.1148/rg.2021200125 34019437 PMC8262179

[33] Budoff MJ Dowe D Jollis JG Gitter M Sutherland J Halamert E Diagnostic performance of 64-multidetector row coronary computed tomographic angiography for evaluation of coronary artery stenosis in individuals without known coronary artery disease: results from the prospective multicenter ACCURACY (assessment by coronary computed tomographic angiography of individuals undergoing invasive coronary angiography) trial. J Am Coll Cardiol. (2008) 52(21):1724–32. 10.1016/j.jacc.2008.07.031 19007693

[34] Tonino PA Fearon WF De Bruyne B Oldroyd KG Leesar MA Ver Lee PN Angiographic versus functional severity of coronary artery stenoses in the FAME study fractional flow reserve versus angiography in multivessel evaluation. J Am Coll Cardiol. (2010) 55(25):2816–21. 10.1016/j.jacc.2009.11.096 20579537

[35] Hänninen H Takala P Korhonen P Oikarinen L Mäkijärvi M Nenonen J Features of ST segment and T-wave in exercise-induced myocardial ischemia evaluated with multichannel magnetocardiography. Ann Med. (2002) 34(2):120–9. 10.1080/07853890252953518 12108575

[36] Alday EA Ni H Zhang C Colman MA Gan Z Zhang H. Comparison of electric- and magnetic-cardiograms produced by myocardial ischemia in models of the human ventricle and torso. PLoS One. (2016) 11(8):e0160999. 10.1371/journal.pone.0160999 27556808 PMC4996509

[37] Park JW Leithäuser B Vrsansky M Jung F. Dobutamine stress magnetocardiography for the detection of significant coronary artery stenoses—a prospective study in comparison with simultaneous 12-lead electrocardiography. Clin Hemorheol Microcirc. (2008) 39(1–4):21–32. 10.3233/CH-2008-1064 18503107

[38] Fenici R Brisinda D. Predictive value of rest magnetocardiography in patients with stable angina. Int Congr Ser. (2007) 1300:737–40. 10.1016/j.ics.2007.02.022

[39] Shin ES Chung JH Park SG Saleh A Lam YY Bhak J Comparison of exercise electrocardiography and magnetocardiography for detection of coronary artery disease using ST-segment fluctuation score. Clin Hemorheol Microcirc. (2019) 73(2):283–91. 10.3233/CH-180485 30775972

[40] Huang X Hua N Tang F Zhang S. Effectiveness of magnetocardiography to identify patients in need of coronary artery revascularization: a cross-sectional study. Cardiovasc Diagn Ther. (2020) 10(4):831–40. 10.21037/cdt-20-121 32968638 PMC7487377

[41] Wu YW Lin LC Tseng WK Liu YB Kao HL Lin MS QTc heterogeneity in rest magnetocardiography is sensitive to detect coronary artery disease: in comparison with stress myocardial perfusion imaging. Acta Cardiol Sin. (2014) 30(5):445–54. 27122818 PMC4834957

[42] Zhang H Ma Z Mi H Jiao J Dong W Yang S Diagnostic value of magnetocardiography to detect abnormal myocardial perfusion: a pilot study. Rev Cardiovasc Med. (2024) 25(10):379. 10.31083/j.rcm2510379 39484136 PMC11522775

[43] Yang S Feng L Zhang M Zhang M Ma Z Zhang H Development and validation of a clinical diagnostic model for myocardial ischaemia in borderline coronary lesions based on optical pumped magnetometer magnetocardiography: a prospective observational cohort study. BMJ Open. (2024) 14(10):e086433. 10.1136/bmjopen-2024-086433 39461859 PMC11529759

[44] Shin ES Lam YY Her AY Brachmann J Jung F Park JW. Incremental diagnostic value of combined quantitative and qualitative parameters of magnetocardiography to detect coronary artery disease. Int J Cardiol. (2017) 228:948–52. 10.1016/j.ijcard.2016.11.165 27912204

[45] Park JW Shin ES Ann SH Godde M Park LS Brachmann J Validation of magnetocardiography versus fractional flow reserve for detection of coronary artery disease. Clin Hemorheol Microcirc. (2015) 59(3):267–81. 10.3233/CH-141912 25480934

[46] Ramesh R Senthilnathan S Satheesh S Swain PP Patel R Ananthakrishna Pillai A Magnetocardiography for identification of coronary ischemia in patients with chest pain and normal resting 12-lead electrocardiogram. Ann Noninvasive Electrocardiol. (2020) 25(3):e12715. 10.1111/anec.12715 31587426 PMC7358824

[47] Hailer B Chaikovsky I Auth-Eisernitz S Schäfer H Van Leeuwen P. The value of magnetocardiography in patients with and without relevant stenoses of the coronary arteries using an unshielded system. Pacing Clin Electrophysiol. (2005) 28(1):8–16. 10.1111/j.1540-8159.2005.09318.x 15660796

[48] Chaikovsky I Hailer B Sosnytskyy V Lutay M Mjasnikov G Kazmirchuk A Predictive value of the complex magnetocardiographic index in patients with intermediate pretest probability of chronic coronary artery disease: results of a two-center study. Coron Artery Dis. (2014) 25(6):474–84. 10.1097/MCA.0000000000000107 24667125

[49] Shin ES Park SG Saleh A Lam YY Bhak J Jung F Magnetocardiography scoring system to predict the presence of obstructive coronary artery disease. Clin Hemorheol Microcirc. (2018) 70(4):365–73. 10.3233/CH-189301 30320564

[50] Cui JG Tian F Miao YH Jin QH Shi YJ Li L Accurate diagnosis of severe coronary stenosis based on resting magnetocardiography: a prospective, single-center, cross-sectional analysis. J Geriatr Cardiol. (2024) 21(4):407–20. 10.26599/1671-5411.2024.04.006 38800545 PMC11112152

[51] Huang X Chen P Tang F Hua N. Detection of coronary artery disease in patients with chest pain: a machine learning model based on magnetocardiography parameters. Clin Hemorheol Microcirc. (2021) 78(3):227–36. 10.3233/CH-200905 33337351

[52] Tantimongcolwat T Naenna T Isarankura-Na-Ayudhya C Embrechts MJ Prachayasittikul V. Identification of ischemic heart disease via machine learning analysis on magnetocardiograms. Comput Biol Med. (2008) 38(7):817–25. 10.1016/j.compbiomed.2008.04.009 18550044

[53] Kangwanariyakul Y Nantasenamat C Tantimongcolwat T Naenna T. Data mining of magnetocardiograms for prediction of ischemic heart disease. EXCLI J. (2010) 9:82–95. 10.17877/DE290R-15805 29255391 PMC5698888

[54] Steinisch M Torke PR Haueisen J Hailer B Gronemeyer D Van Leeuwen P Early detection of coronary artery disease in patients studied with magnetocardiography: an automatic classification system based on signal entropy. Comput Biol Med. (2013) 43(2):144–53. 10.1016/j.compbiomed.2012.11.014 23260570

[55] Rong T Shulin Z Xiao H Minfang T Jian M Shixin M Magnetocardiography-based ischemic heart disease detection and localization using machine learning methods. IEEE Trans Biomed Eng. (2019) 66(6):1658–67. 10.1109/TBME.2018.2877649 30369432

[56] Ashokprabhu N Ziada K Daher E Cho L Schmidt CW Roca Y Evaluation of coronary microvascular dysfunction using magnetocardiography: a new application to an old technology. Am Heart J Plus. (2024) 44:100424. 10.1016/j.ahjo.2024.100424 39108843 PMC11301233

[57] Wang Y Jia H Song J. Accurate classification of non-ischemic cardiomyopathy. Curr Cardiol Rep. (2023) 25(10):1299–317. 10.1007/s11886-023-01944-0 37721634 PMC10651539

[58] Brala D Thevathasan T Grahl S Barrow S Violano M Bergs H Application of magnetocardiography to screen for inflammatory cardiomyopathy and monitor treatment response. J Am Heart Assoc. (2023) 12(4):e027619. 10.1161/JAHA.122.027619 36744683 PMC10111485

[59] Kawakami S Takaki H Hashimoto S Kimura Y Nakashima T Aiba T Utility of high-resolution magnetocardiography to predict later cardiac events in nonischemic cardiomyopathy patients with normal QRS duration. Circ J. (2016) 81(1):44–51. 10.1253/circj.CJ-16-0683 27853097

[60] Schirdewan A Gapelyuk A Fischer R Koch L Schütt H Zacharzowsky U Cardiac magnetic field map topology quantified by Kullback-Leibler entropy identifies patients with hypertrophic cardiomyopathy. Chaos. (2007) 17(1):015118. 10.1063/1.2432059 17411275

[61] Iwakami N Aiba T Kamakura S Takaki H Furukawa TA Sato T Identification of malignant early repolarization pattern by late QRS activity in high-resolution magnetocardiography. Ann Noninvasive Electrocardiol. (2020) 25(4):e12741. 10.1111/anec.12741 31955494 PMC7358799

[62] Lombardi G Sorbo AR Guida G La Brocca L Fenici R Brisinda D. Magnetocardiographic classification and non-invasive electro-anatomical imaging of outflow tract ventricular arrhythmias in recreational sport activity practitioners. J Electrocardiol. (2018) 51(3):433–9. 10.1016/j.jelectrocard.2018.02.004 29486898

[63] Korhonen P Husa T Tierala I Väänänen H Mäkijärvi M Katila T QRS Duration in high-resolution methods and standard ECG in risk assessment after first and recurrent myocardial infarctions. Pacing Clin Electrophysiol. (2006) 29(8):830–6. 10.1111/j.1540-8159.2006.00448.x 16922998

[64] Korhonen P Tierala I Simelius K Väänänen H Mäkijärvi M Nenonen J Late QRS activity in signal-averaged magnetocardiography, body surface potential mapping, and orthogonal ECG in postinfarction ventricular tachycardia patients. Ann Noninvasive Electrocardiol. (2002) 7(4):389–98. 10.1111/j.1542-474X.2002.tb00190.x 12431319 PMC7027709

[65] Guida G Sorbo AR Fenici R Brisinda D. Predictive value of unshielded magnetocardiographic mapping to differentiate atrial fibrillation patients from healthy subjects. Ann Noninvasive Electrocardiol. (2018) 23(6):e12569. 10.1111/anec.12569 29947446 PMC6931487

[66] Her AY Shin ES Zhou Q Wierzbinski J Vidal-Lopez S Saleh A Magnetocardiography detects left atrial dysfunction in paroxysmal atrial fibrillation. Clin Hemorheol Microcirc. (2019) 72(4):353–63. 10.3233/CH-180528 30958336

[67] Jurkko R Mäntynen V Tapanainen JM Montonen J Väänänen H Parikka H Non-invasive detection of conduction pathways to left atrium using magnetocardiography: validation by intra-cardiac electroanatomic mapping. Europace. (2009) 11(2):169–77. 10.1093/europace/eun335 19074785

[68] Ito Y Shiga K Yoshida K Ogata K Kandori A Inaba T Development of a magnetocardiography-based algorithm for discrimination between ventricular arrhythmias originating from the right ventricular outflow tract and those originating from the aortic sinus cusp: a pilot study. Heart Rhythm. (2014) 11(9):1605–12. 10.1016/j.hrthm.2014.05.032 24887136

[69] Aita S Ogata K Yoshida K Inaba T Kosuge H Machino T Noninvasive mapping of premature ventricular contractions by merging magnetocardiography and computed tomography. JACC Clin Electrophysiol. (2019) 5(10):1144–57. 10.1016/j.jacep.2019.06.010 31648739

[70] Wacker-Gussmann A Eckstein GK Strasburger JF. Preventing and treating torsades de pointes in the mother, fetus and newborn in the highest risk pregnancies with inherited arrhythmia syndromes. J Clin Med. (2023) 12(10):3379. 10.3390/jcm12103379 37240485 PMC10219273

[71] Wacker-Gussmann A Strasburger JF Wakai RT. Contribution of fetal magnetocardiography to diagnosis, risk assessment, and treatment of fetal arrhythmia. J Am Heart Assoc. (2022) 11(15):e025224. 10.1161/JAHA.121.025224 35904205 PMC9375504

[72] Yuan SM. Fetal arrhythmias: surveillance and management. Hellenic J Cardiol. (2019) 60(2):72–81. 10.1016/j.hjc.2018.12.003 30576831

[73] Strand SA Strasburger JF Wakai RT. Fetal magnetocardiogram waveform characteristics. Physiol Meas. (2019) 40(3):035002. 10.1088/1361-6579/ab0a2c 30802886 PMC6449176

[74] Stingl K Paulsen H Weiss M Preissl H Abele H Goelz R Development and application of an automated extraction algorithm for fetal magnetocardiography—normal data and arrhythmia detection. J Perinat Med. (2013) 41(6):725–34. 10.1515/jpm-2013-0031 23828424

[75] Donofrio MT Moon-Grady AJ Hornberger LK Copel JA Sklansky MS Abuhamad A Diagnosis and treatment of fetal cardiac disease: a scientific statement from the American Heart Association. Circulation. (2014) 129(21):2183–242. 10.1161/01.cir.0000437597.44550.5d 24763516

[76] Batra AS Silka MJ Borquez A Cuneo B Dechert B Jaeggi E Pharmacological management of cardiac arrhythmias in the fetal and neonatal periods: a scientific statement from the American Heart Association: endorsed by the pediatric & congenital electrophysiology society (PACES). Circulation. (2024) 149(10):e937–e52. 10.1161/CIR.0000000000001206 38314551

[77] Kiefer-Schmidt I Lim M Wacker-Gussmann A Ortiz E Abele H Kagan KO Fetal magnetocardiography (fMCG): moving forward in the establishment of clinical reference data by advanced biomagnetic instrumentation and analysis. J Perinat Med. (2012) 40(3):277–86. 10.1515/jpm.2011.139 22505507

[78] Van Norstrand DW Ackerman MJ. Sudden infant death syndrome: do ion channels play a role? Heart Rhythm. (2009) 6(2):272–8. 10.1016/j.hrthm.2008.07.028 18823823 PMC3940066

[79] Cuneo BF Strasburger JF Yu S Horigome H Hosono T Kandori A In utero diagnosis of long QT syndrome by magnetocardiography. Circulation. (2013) 128(20):2183–91. 10.1161/CIRCULATIONAHA.113.004840 24218437 PMC3831174

[80] Kiefer-Schmidt I Lim M Preissl H Draganova R Weiss M Abele H Fetal magnetocardiography (fMCG) to monitor cardiac time intervals in fetuses at risk for isoimmune AV block. Lupus. (2014) 23(9):919–25. 10.1177/0961203314527364 24639473

[81] Zhao H Cuneo BF Strasburger JF Huhta JC Gotteiner NL Wakai RT. Electrophysiological characteristics of fetal atrioventricular block. J Am Coll Cardiol. (2008) 51(1):77–84. 10.1016/j.jacc.2007.06.060 18174041 PMC3296565

[82] Cuneo BF Bitant S Strasburger JF Kaizer AM Wakai RT. Assessment of atrioventricular conduction by echocardiography and magnetocardiography in normal and anti-Ro/SSA-antibody-positive pregnancies. Ultrasound Obstet Gynecol. (2019) 54(5):625–33. 10.1002/uog.20245 30784137 PMC6699937

[83] Wiggins DL Strasburger JF Gotteiner NL Cuneo B Wakai RT. Magnetophysiologic and echocardiographic comparison of blocked atrial bigeminy and 2:1 atrioventricular block in the fetus. Heart Rhythm. (2013) 10(8):1192–8. 10.1016/j.hrthm.2013.04.020 23619035 PMC3757956

